# Three Cases of Hepatocellular Carcinoma With Massive Macrovascular Invasion Successfully Treated With Radiotherapy

**DOI:** 10.7759/cureus.18624

**Published:** 2021-10-09

**Authors:** Yuichiro Tsurugai, Atsuya Takeda, Naoko Sanuki, Takahisa Eriguchi, Masayuki Ueno

**Affiliations:** 1 Radiation Oncology Center, Ofuna Chuo Hospital, Kamakura, JPN; 2 Department of Gastroenterology and Hepatology, Kurashiki Central Hospital, Kurashiki, JPN

**Keywords:** multidisciplinary treatments, stereotactic body radiotherapy, radiotherapy, inferior vena cava tumor thrombosis, portal vein tumor thrombosis, macrovascular invasion, hepatocellular carcinoma

## Abstract

In clinical practice, the treatment approach for hepatocellular carcinoma (HCC) with macrovascular invasion (MVI) is determined on a case-by-case basis. The common management options include systemic and local therapies, although the former is the more widely accepted approach. We present three cases of HCC with MVI successfully treated with radiotherapy. The first patient was a 62-year-old man with Child-Pugh A cirrhosis who had a 5.7-cm treatment-naïve HCC invading the bilateral branches of the portal vein. Stereotactic body radiotherapy (SBRT) was administered, with no evidence of recurrence observed at the 24-month follow-up. The second patient was an 81-year-old man with Child-Pugh A cirrhosis who had a 3.8-cm HCC invading the inferior vena cava (IVC). Transcatheter chemoembolization performed one month earlier had been ineffective, and the tumor had grown rapidly. SBRT was administered, and no evidence of recurrence was observed up to his death from pneumonia 24 months after the treatment initiation. The third patient was a 72-year-old man with Child-Pugh A cirrhosis who had a 6.7-cm treatment-naïve HCC with portal vein tumor thrombosis (PVTT) from the main trunk to the secondary branches of both lobes. PVTT was treated with hypofractionated radiotherapy, while the primary HCC and intrahepatic recurrent lesions were subsequently treated with hepatic arterial infusion chemotherapy (HAIC) and five rounds of ablation. Six months after the last ablation (48 months after initial therapy), no evidence of recurrence was observed. Our cases illustrate that radiotherapy leads to the successful treatment of HCC with MVI.

## Introduction

For hepatocellular carcinoma (HCC) with macrovascular invasion (MVI), systemic therapy has been the most widely accepted treatment approach [1-5]. However, the outcomes remain unsatisfactory despite the introduction of novel molecularly targeted agents besides sorafenib [6,7]. Multiple local therapies have been described in several guidelines as treatment options for HCC with MVI. The recent Japanese guidelines [2] list transcatheter arterial chemoembolization (TACE), hepatic resection, and hepatic arterial infusion chemotherapy (HAIC), while the American Association for the Study of Liver Diseases (AASLD) guidelines [3] and several Asian guidelines [4,5] list radiotherapy along with the above-mentioned treatment modalities. However, no robust evidence currently exists regarding the superiority of these treatment modalities for HCC patients with MVI. It has therefore been recommended that treatment selection should be made on a case-to-case basis, by considering the tumor size and location, liver function, comorbidities, and the general condition of the patient [2].

Advances in radiotherapy, including stereotactic body radiotherapy (SBRT) and intensity-modulated radiotherapy, have enabled the targeting of high radiation doses on lesions while reducing the exposure to surrounding normal tissues. Several multicenter prospective studies on early-stage HCC patients have reported excellent outcomes following SBRT, with a three-year local control rate of >90% and minimal toxicity [8,9]. Additionally, radiotherapy has a unique advantage over other local treatments, given its safety in treating lesions adjacent to large blood vessels and bile ducts [10,11]. Favorable therapeutic outcomes have also been reported in HCC patients with MVI [12-14].

In this article, we present three cases of HCC with MVI successfully managed with radiotherapy, and discuss the role of radiotherapy as part of multidisciplinary treatment strategies.

## Case presentation

SBRT monotherapy

**Figure 1:**
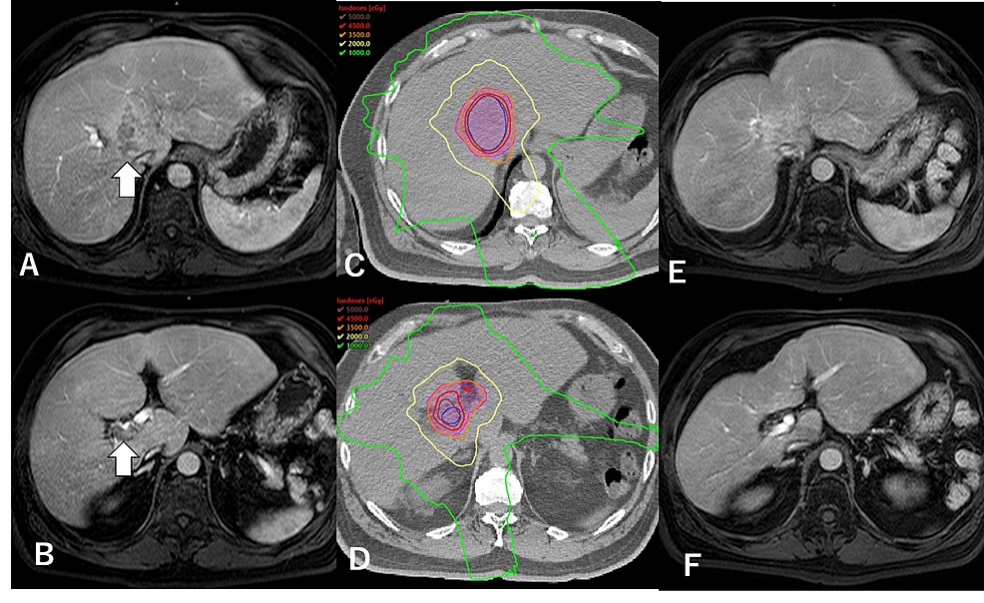
Case 1 – SBRT monotherapy A, B: pretreatment MRI of the liver demonstrating hepatocellular carcinoma invading bilateral portal vein invasion (arrows). C, D: axial images of the dose distribution of radiotherapy. SBRT was performed with 35 Gy in five fractions prescribed to enclose the planning target volume with a 60% isodose line of the maximal dose equated to the prescribed dose. E, F: the latest MRI with no recurrence 24 months after SBRT. The portal vein was kept flowing MRI: magnetic resonance imaging: SBRT: stereotactic body radiotherapy

A 62-year-old man with nonalcoholic fatty liver disease and Child-Pugh 5A cirrhosis [albumin-bilirubin (ALBI) grade 1] presented with a 5.7 x 3.1-cm treatment-naïve HCC invading the bilateral branches of the portal vein (Figures 1A, 1B). His serum alpha-fetoprotein (AFP) and des-γ-carboxy prothrombin (PIVKA-II) levels were 3,357.5 ng/mL and 135 mAU/mL, respectively. His hepatologist consulted the radiation oncologist, who proposed that SBRT could be one of the initial treatment options since the lesion distance from the gastrointestinal tract was sufficient for the safe delivery of radical doses. After discussion and obtaining informed consent, SBRT with 35 Gy in five fractions was administered to enclose the planning target volume with a 60% isodose line of the maximal dose equated to the prescribed dose (Figures 1C, 1D). Within one month, the patient's AFP and PIVKA-II levels decreased to 278 ng/mL and 33 mAU/mL, respectively; the portal vein tumor thrombosis (PVTT) shrank, and his liver function remained preserved. At the 24-month follow-up, his AFP and PIVKA-II levels were found to have reduced at 4.7 ng/mL and 24 mAU/mL, respectively, with no evidence of recurrence observed on MRI (Figures 1E, 1F). His liver function remained at Child-Pugh 5A cirrhosis (ALBI grade 1).

Salvage SBRT for lesion unresponsive to TACE

**Figure 2:**
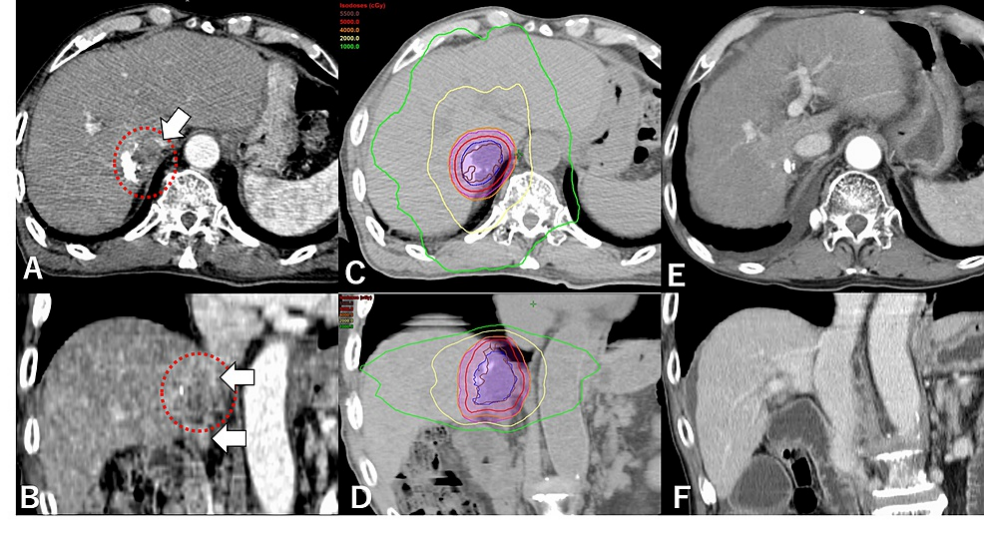
Case 2 – salvage SBRT for lesion unresponsive to TACE A, B: treatment planning CT of the liver demonstrating hepatocellular carcinoma (dotted line) invading the inferior vena cava (IVC) (arrows). C, D: axial and coronal images of the dose distribution of radiotherapy. SBRT was performed with 40 Gy in five fractions prescribed to enclose the planning target volume with a 70% isodose line of the maximal dose equated to the prescribed dose. E, F: the latest CT with no recurrence 22 months after SBRT. The IVC was kept flowing. The patient died of pneumonia two months after this scan SBRT: stereotactic body radiotherapy; TACE: transcatheter arterial chemoembolization; CT: computed tomography

An 81-year-old man with chronic hepatitis C infection and Child-Pugh 5A cirrhosis (ALBI grade 1) presented with a 3.8-cm HCC invading the inferior vena cava (IVC). TACE performed one month earlier had been ineffective, and the tumor had rapidly grown (Figures 2A, 2B). His serum AFP and PIVKA-II levels were 114.8 ng/mL and 3,570 mAU/mL, respectively. SBRT was hence administered with 40 Gy in five fractions to enclose the planning target volume with a 70% isodose line of the maximal dose equated to the prescribed dose (Figures 2C, 2D). Within one month, his AFP and PIVKA-II levels decreased to 14.4 ng/mL and 249 mAU/mL, respectively; the IVC lesion disappeared, and his liver function remained preserved. Until his death from pneumonia 24 months after SBRT initiation, there was no evidence of recurrence on CT (Figures 2E, 2F).

Hypofractionated radiotherapy followed by multidisciplinary treatments

**Figure 3:**
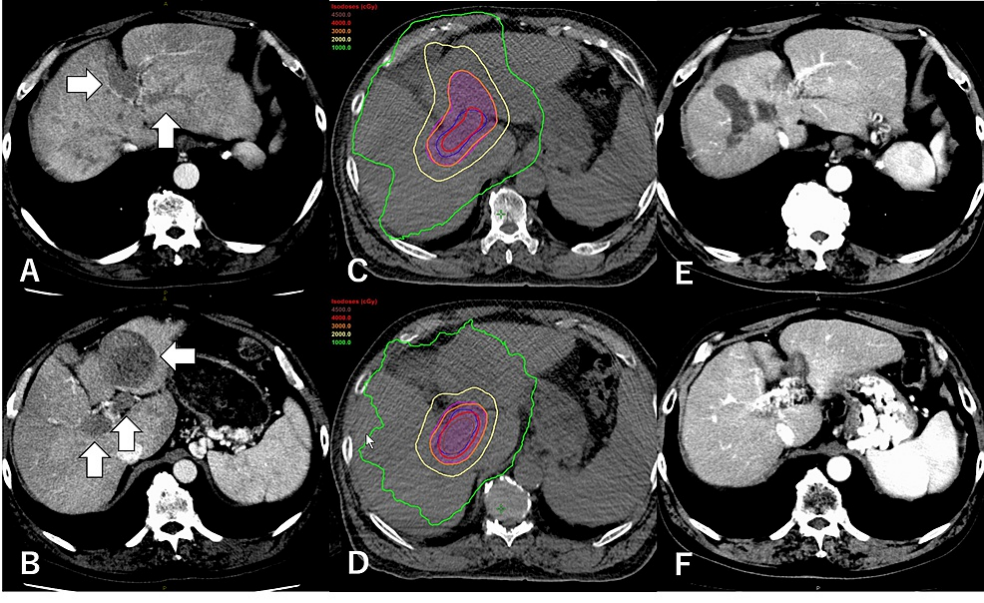
Case 3 – hypofractionated radiotherapy followed by multidisciplinary treatments A, B: pretreatment CT of the liver demonstrating hepatocellular carcinoma with massive portal vein tumor thrombosis from the main trunk to the secondary branches of both lobes (arrows). C, D: axial images of the dose distribution of radiotherapy. Hypofractionated radiotherapy was performed with 30 Gy in 10 fractions prescribed to enclose the planning target volume with a 60% isodose line of the maximal dose equated to the prescribed dose. E, F: the latest CT scan with no recurrence after 48 months after radiotherapy. Portal vein invasion has been thrombosed and occluded, with collateral blood vessels being formed CT: computed tomography

A 72-year-old man with alcoholic liver disease and Child-Pugh 6A cirrhosis (ALBI grade 2) presented to another hospital with a 6.7-cm treatment-naïve HCC with a massive PVTT from the main trunk to the secondary branches of both lobes (Figures 3A, 3B). His serum AFP and PIVKA-II levels were 935.0 ng/mL and 352 mAU/mL, respectively. Following the refusal of all recommended treatment options such as conservative treatment, HAIC, and sorafenib monotherapy, the physician consulted our hospital to suggest an indication for radiotherapy. After shared decision making, the PVTT was treated with hypofractionated radiotherapy with 30 Gy in 10 fractions to enclose the planning target volume with a 60% isodose line of the maximal dose equated to the prescribed dose (Figures 3C, 3D). The treatment intensity was reduced due to concerns of gastrointestinal toxicity. Within two months, his AFP and PIVKA-II levels decreased to 81 ng/mL and 14 mAU/mL, respectively; the PVTT shrank, and his liver function remained preserved. Three months after SBRT, HAIC was initiated at two-month intervals. Imaging examinations conducted nine months after SBRT revealed intrahepatic recurrence, although the PVTT continued to shrink. The primary HCC and intrahepatic recurrent lesions were subsequently treated with five rounds of ablation over a 14-month period. At the latest follow-up conducted six months after the last ablation (48 months after initial therapy), his AFP and PIVKA-II levels were found to be reduced to <2 ng/mL and 19 mAU/mL, respectively. No evidence of recurrence was observed on CT (Figures 3E, 3F), and his liver function remained at Child-Pugh 6A cirrhosis (ALBI grade 2).

## Discussion

Our cases showed that radiotherapy can contribute to the successful management of HCC with MVI. Two patients achieved long-term recurrence-free survival with SBRT, while the other achieved long-term survival with hypofractionated radiotherapy followed by multidisciplinary treatments.

While several studies have reported the efficacy of local therapies for MVI, including resection, HAIC, and TACE, the outcomes have not been satisfactory, and the treatment indications are often limited. Propensity score matching analysis of a Japanese nationwide survey showed significantly longer median survival following treatment with liver resection (LR) than with non-LR treatments in patients with Child-Pugh A cirrhosis and PVTT of the first or peripheral branches (2.5 years vs. 1.6 years, p<0.001) [15]. However, the resection rate of HCC with PVTT in the first branch was limited to 24%, due to technical demands and excessive invasiveness depending on the location and extent of the tumor thrombus. HAIC or TACE alone, or in combination with systemic therapy, have only modestly improved the prognosis of patients with unresectable MVI [16,17]. In the sub-analysis of a phase 3 trial, combination therapy with HAIC and sorafenib resulted in only a statistically marginal survival benefit compared with sorafenib monotherapy in patients with main portal vein invasion (11.4 months vs. 6.5 months, p=0.050) [16]. In a meta-analysis of HCC with PVTT, a limited response rate of 19% with TACE has been reported [18]. In a propensity score analysis, TACE was only associated with a four-month survival benefit when compared with conservative treatment (11 months vs. seven months, p=0.002) [19].

As with early-stage HCC, radiotherapy can also be performed with curative intent for HCC with MVI since vascular invasion itself does not cause treatment-related toxicities. Thus, high local control with relatively less invasiveness can be expected, particularly in the presence of conditions such as preserved liver function, small to medium-sized tumors, or sufficient distance from the gastrointestinal tract. Therefore, radiotherapy can be a reasonable and effective local treatment option for HCC with MVI. A phase I and II study on SBRT for advanced HCC, in which 55% of the patients had MVI, has reported encouraging outcomes with a one-year local control rate of 87% and a median overall survival of 17.0 months [12]. Munoz-Schuffenegger et al. retrospectively investigated SBRT in 128 cases of HCC with MVI, 66% of which involved the first branch or main trunk of the portal vein, and showed a one-year local control rate of 87.4%, median survival of 18.3 months, and only four cases of gastrointestinal bleeding [13].

Radiotherapy in combination with other modalities represents a reasonable first-line treatment option for HCC patients with MVI [13,20] due to its high efficacy and prompt tumor shrinkage effects. This approach may also improve the effectiveness of subsequent treatment. In a randomized clinical trial comparing the combination of TACE and radiotherapy (TACE-RT) with sorafenib monotherapy in HCC patients with MVI, the overall survival in the TACE-RT group was significantly longer than that in the sorafenib group (12.7 vs. 9.9 months, p=0.04). The authors recommended TACE-RT rather than sorafenib as initial therapy, not only because TACE-RT was associated with better outcomes, but also because 91% of patients in the sorafenib group were eventually treated with TACE-RT during disease progression [20]. In the aforementioned retrospective study of HCC with MVI [13], patients who received SBRT followed by sorafenib had a median survival time of 37.9 months. In addition, tumor shrinkage was shown in 70% of HCC patients with MVI within one month of SBRT [14], which allowed for prompt subsequent treatment.

## Conclusions

We presented three cases in which radiotherapy contributed to the successful management of HCC with MVI. Radiotherapy may therefore play an important role in the treatment of HCC with MVI. The incorporation of radiotherapy into multidisciplinary treatment strategies, whether with radical or semi-radical intent, would improve the overall prognosis of HCC.
